# One simple question detects motion sickness susceptibility in migraine patients

**DOI:** 10.1016/j.bjorl.2023.101382

**Published:** 2023-12-19

**Authors:** Gülden Akdal, Pınar Özçelik, Birgül Balcı, Gábor Michael Halmágyi, Belgin Ünal

**Affiliations:** a Dokuz Eylül University, Faculty of Medicine, Department of Neurology, Izmir, Turkey; b Dokuz Eylül University, Institute of Health Sciences, Department of Neuroscience, Izmir, Turkey; c Bezmialem Vakif University, Faculty of Medicine, Department of Neurology, Istanbul, Turkey; d Dokuz Eylül University, School of Physiotherapy and Rehabilitation, Izmir, Turkey; e University of Sydney, Central Clinical School, Sydney, Australia; f Dokuz Eylül University, Faculty of Medicine, Department of Public Health, Izmir, Turkey

**Keywords:** Motion sickness, Vestibular migraine, Migraine, Vertigo

## Abstract

**Objective:**

To find out if motion sickness susceptibility (MSS) of vestibular migraine (VM) patients and migraine only (MO) patients can be reliably detected with a single simple question: “Can you read while travelling in a car without getting motion sick?”.

**Method:**

Ninety-two definite VM and 58 MO patients and 74 healthy control (HC) subjects were asked about their MSS and about being able to read while riding in a car without becoming motion sick. A Motion Sickness Susceptibility Questionnaire (MSSQ-Short) including childhood (MSA), adulthood (MSB) and total (MST) parts was also administered to all participants. ROC curves of MSSQ-Short were prepared for “not being able to read in a car” as the gold standard.

**Results:**

Mean MSA scores were significantly higher in both VM and MO patients than in HCs (*p* < 0.001), but their scores were not significantly different (*p* = 0.171). Mean MSB and MST scores were significantly higher in VM than in MO patients (*p* < 0.001) and both VM and MO patients had significantly higher scores than HCs (*p* < 0.001). MSA scores were significantly higher than MSB scores in MO patients (*p* < 0.001). All sections of the questionnaire were associated with high area-under-curve values for MSS detected by the question about being able to read in a car without becoming motion sick.

**Conclusion:**

We propose that all migraine patients could have the same level of MSS in childhood but MO patients are able to compensate over years, but VM patients are not. A quick way to determine MSS is to ask about the ability to read without becoming motion sickness while riding a car.

## Highlights

• Motion sickness is very common in patients with vestibular migraine.
• Motion sickness questionnaires are time-consuming for patient and doctor.
• A positive answer to just one simple question gives comparable results.
• The question: While riding in a car or bus, can you read without getting motion sick?

## Introduction

Motion sickness is provoked by passive motion of self, or of the visual environment, or of both, and commonly occurs while riding in cars, buses, boats, planes etc.^1^, ^2^

Symptomatic motion sickness susceptibility (MSS) is common in migraine patients. Even between attacks, migraine patients^3^, ^4^, ^5^ especially those with vestibular migraine (VM)^6^, ^7^ are particularly susceptible to motion sickness; they can have it when they try to read while riding in a car or bus.^8^, ^9^

The aim of our study was to determine if being unable to read while riding in a car without developing motion sickness is a useful clinical approximation of formal MSS questionnaire scores.^10^

## Methods

We studied 92 definite VM patients^11^ (ages 21–61 years) who did not have history of other neurological disorders and had normal audiogram, 58 migraine patients without a history of vertigo (MO)^12^ (aged 19–60 years) and 74 healthy control subjects (HC) (aged 19–81 years). VM patients were recruited from our neuro-otology outpatient clinic, MO patients and HCs by advertisement. HCs had normal neurological examination and did not have a history of headache or vertigo. Minimum number of participants in each group was estimated at 52, assuming medium effect size (f = 0.25) and 80% power).^13^ All participants gave their informed consent and local ethical committee approved the study.

Motion sickness was questioned by two methods.

(A) This 2-item questionnaire: Question 1: Could you read while riding in a car or bus without becoming motion sick? Question 2: Do you have motion sickness while riding in a car or bus?
(B) MSSQ Short questionnaire which investigates motion sickness with each mode of transportation. This questionnaire is in two sections: A (childhood-MSA) and B (adulthood-MSB). Each section consists of 9-items graded 0–3, with higher scores signifying higher motion sickness susceptibility. The score given depends on how much sickness or nausea is felt during travel. Sum of MSA and MSB forms MSSQ Short-Total (MST).^10^

### Statistics

Shapiro–Wilk test was used to check whether MSSQ-Short scale were normally distributed between groups. One way analysis of variance (One-way ANOVA) with Bonferroni Correction was used to determine whether demographical variables and MSSQ-Short all forms were different between VM patients, MO patients and HCs.

Paired *t*-test was used to compare A and B sections of MSSQ-Short questionnaire scores. ROC curves of MSSQ-Short questionnaire were prepared for “not being able to read in the car” as a gold standard. Cut-off points for MSA, MSB and MST were determined using sensitivity, specifity estimates from ROC analyses. Positive and negative predictive values and 95% CIs were calculated using Openepi (http://www.openepi.com/DiagnosticTest/DiagnosticTest.htm). All data were analyzed using SPSS 15.0 version 15 programme.

## Results

There were no significant age (*p* = 0.308) or gender (*p* = 0.831) differences between VM, MO patients and HCs.

VM and MO patients were both more likely than HCs (*p* < 0.001) to report motion sickness (Question 1) and they were also more likely than HCs (*p* < 0.001) to report being unable to read in a moving car without becoming motion sick (Question 2) (Table 1).

**Table 1:** Comparison of the demographic features and the answers of the 2-item questionnaire in VM, MO and HCs.

	VM (n = 92)	MO (n = 58)	HCs (n = 74)	*p*-Value
Age (year)	41.4 ± 9.5	38.3 ± 10.5	39.2 ± 16.2	0.308^a^
Gender (female/male)	F:78/M:14	F:50/M:8	F:61/M:13	0.831^b^
Q1: Do you have motion sickness while riding in a car or bus? (Yes)	73 (79.3%)	25 (43.9%)	7 (9.5%)	**<0.001**^b^
Q2: Could you read while riding in a car or bus without becoming motion sick? (No)	79 (85.6%)	29 (50%)	11 (14.9%)	**<0.001**^b^

VM, vestibular migraine; MO, migraine without vertigo; HCs, healthy controls; n, number in groups.

Signifcant value is highlighted in bold, *p* < 0.05.

a Oneway Anova test.

b Chi-Square test.

Mean scores of both VM and MO patients were significantly higher on the MSA section than of HCs (*p* < 0.001) but the VM patients’ scores were not significantly different to MO patients’ scores of (*p* = 0.171).

On the other hand, mean MSB and MST scores were different between patient groups; VM patients had higher scores than MO patients (*p* < 0.001), and both had significantly higher scores than the HCs (*p* < 0.001) (Table 2).

**Table 2:** Comparison of MSSQ-Short results between VM, MO and HCs.

	VM (n = 92)	MO (n = 58)	HCs (n = 74)	VM & MO^a^*p-*values	VM & HCs^a^*p-*values	MO & HCs^a^*p-*values	VM & MO & HCs^b^*p-*values
MSA	15.0 ± 9.8	12.3 ± 9.5	2.6 ± 4.5	0.171	**<0.001**	**<0.001**	**<0.001**
MSB	14.8 ± 8.7	6.8 ± 6.2	2.3 ± 2.9	**<0.001**	**<0.001**	**<0.001**	**<0.001**
MST	29.8 ± 16.4	19.1 ± 14.6	4.8 ± 6.3	**<0.001**	**<0.001**	**<0.001**	**<0.001**

VM, vestibular migraine; MO, migraine without vertigo; HCs, healthy controls; MSA, MSSQ-Short for childhood (A); MSB, MSSQ-Short for adulthood (B); MST, MSSQ-Short Total.

Signifcant value is highlighted in bold, *p* < 0.05.

a Post-hoc test, Bonferonni correction.

b One way Anova test.

Mean MSB scores were lower than MSA scores in MO patients (*p* < 0.001), but did not differ in VM patients (*p* = 0.81) or in HCs (*p* = 0.48) (Table 2).

The possible cutoff scores for motion sickness in the MSA is 4.25 (Sensitivity = 83.3%, 95% CI 75.2%‒89.1%) and for MSB 5.25 (Sensitivity = 89.8%, 95% CI 82.6%–94.2%). We suggest that the cutoff point MST scores is 8.50 (Sensitivity = 91.6%, 95% CI 84.9%–95.6%) (Table 3).

**Table 3:** Validity measures for the three best cut-off points of motion sickness, analyses for all scores of MSSQ-Short.

	AUC values (95% CI)	Cut-off score	Sensitivity	Specificity	PPV	NPV
MSA	0.80^a^ (0.74‒0.86)	5.25	83.3%	70.2%	74.4%	80.2%
MSB	0.90^a^ (0.86‒0.94)	4.25	89.8%	74.0%	78.2%	87.5%
MST	0.87^a^ (0.83‒0.92)	8.50	91.7%	70.2%	76.2%	89.0%

PPV, positive predictive value; NPV, negative predictive value; CI, confidence interval; MSA, MSSQ-Short for childhood (A); MSB, MSSQ-short for adulthood (B); MST, MSSQ-Short Total score.

a *p* < 0.001.

**Figure 1:**
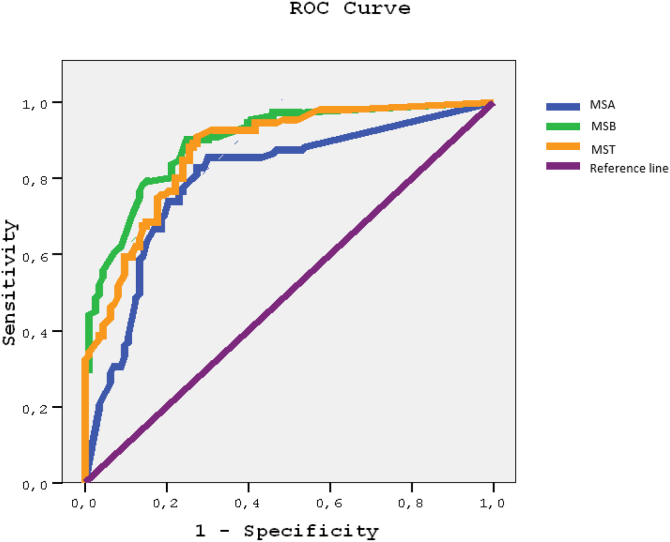
ROC curves of MSSQ scores (MSA, MSB, MST) with the response to the “reading in the car while riding” question as the gold standard.

ROC curves and AUC values were derived (Fig. 1) with the corresponding 95% confidence interval (95% CI) using “not able read while riding in a car” as the gold standard. Graphs moving toward the upper left corner represent an increasing AUC value with progressively higher rates of true positives (sensitivity) and higher rates of true negatives (specificity). Higher AUC of each section of MSSQ-Short indicates a high diagnostic accuracy for motion sickness. MSB score had the hightest AUC (AUC = 0.909, 95% CI 0.86–0.94), followed by the MST score (AUC = 0.87, 95% CI 0.83–0.92) and then MSA score (AUC = 0.80, 95% CI 0.74–0.86).

## Discussion

Here we found that both VM and MO patients have much higher MSA, MSB and MST scores than HCs and that VM patients have significantly higher MSB and MST (but not MSA) scores than MO patients.

A possible explanation for this is that MO patients are able to adapt to motion sickness, whereas VM patients are not. This explanation is supported by our previous study comparing MSSQ scores with Dynamic Gait Index (DGI) and Dizziness Handicap (DHI) in VM and MO patients.^14^ Higher MSSQ scores were correlated with worse DGI and DHI scores supporting the notion of MSS as an indicator of vestibular hypersensitivity.

Around 2/3 of the population, will at some time, experience carsickness^15^ and with certain provocative manoeuvres almost anyone can be made motion sick.^16^ On the other hand, migraine patients, especially VM patients have decreased thresholds for angular acceleration perception and have higher MSS than HCs.^17^ Hypersensitivity of the vestibular system could be a mechanism for MSS in VM patients.^4^ Migraine, vertigo and motion sickness could share pathophysiology^3^ and migraine prevention treatment could reduce vertigo handicap and MSS of VM patients.^18^

Although an online survey of 277 unselected students, found no significant correlation between MSS scores in what was considered to be VM or MO,^19^ this negative finding might be the result of the methodology; an online survey in contrast to the face-to-face medical consultation in our study.

In order to read clearly while in a travelling car, it is necessary to suppress vestibulo-ocular reflexes triggered by vehicle motion.^16^ When a person’s vestibular system is sensitive,^17^ it is difficult read while riding in a car without developing motion sickness, perhaps because of the visual-vestibular conflict.^1^

We agree^14^ that it is useful to ask dizzy patients about MSS (2) but there is not always time in a busy dizzy clinic to administer a full MSS questionnaire. We routinely ask our patients if they consider if they are susceptible to motion sickness and also if they are able to read when travelling in a car or bus without becoming motion sick.

Here 6 of 92 VM patients (mean MSSQ total score of 29.8), 4 of 58 MO patients (mean MSSQ total score of 19.1) and 4 of 74 HCs (mean MSSQ total score of 4.8) denied being susceptible to motion sickness but nonetheless admitted to being unable to read while travelling without developing motion sickness.

The relevance of our observations for the clinician is that a long history of MSS preceding the development of recurrent spontaneous vertigo, can be reliably detected with a single question about reading while riding in a car.

## Conclusion

Being unable to read while riding without getting motion sick is a useful indicator of vestibular migraine.

## Funding

This study was not supported by any funding.

## Conflicts of interest

The authors declare no conflicts of interest.
